# Lenacapavir’s success: Revitalizing antiviral drug discovery

**DOI:** 10.1080/21505594.2025.2497902

**Published:** 2025-05-02

**Authors:** Daniel Miranda, David Jesse Sanchez

**Affiliations:** Pharmaceutical Sciences Department, Western University of Health Sciences, Pomona, CA, USA

**Keywords:** HIV, drug target, drug discovery, lenacapavir, capsid inhibitor, antiviral

Lenacapavir has emerged as a potential game-changer for both HIV treatment and prophylaxis. This new antiviral is a first-in-class drug targeting the HIV capsid protein – a viral target that up until the work leading to lenacapavir has not often been included in the drug-target conversations regarding HIV antivirals [1]. Lenacapavir is a small-molecule inhibitor that disrupts HIV-1 replication by binding to the capsid protein and interfering with different steps in the viral replication cycle, including capsid assembly and disassembly. The outcomes of the lenacapavir clinical trials show tremendous promise: 100% effective in preventing HIV in cisgender women [2], reduced HIV incidence by 96% in a diverse cohort of cisgender men, transgender, and nonbinary individuals [3], and virologic suppression in heavily treatment-experienced adults with multidrug-resistant (MDR) HIV-1 infection [4,5]. Complemented by a pandemic that has no approved vaccination nor long-term cure, lenacapavir represents a potential path to global control of HIV infection.

The development of lenacapavir is a prime example of taking translational basic research on virus replication beyond the validated targets that are focused on in the current antiviral standard of care. From cryo-EM studies into capsid structure to more detailed X-ray crystallography, the initial studies into the basic structural biology of the capsid protein set up a path toward developing lenacapavir [6,7]. Adjacently, development of capsid inhibitors such as GS-CA1 provided strong preclinical support of this path toward a new antiviral [8], and the successive development of the derivative GS-6207, later granted the nonproprietary name lenacapavir through the International Nonproprietary Name (INN) process, added long-acting bioavailability properties, enabling up to six months between administrations. The longer-acting enhancement of this capsid inhibitor also represents a significant advancement in reducing the burden of frequent dosing, thereby bolstering the potential for patient adherence and outcomes [9].

## Antiviral drug discovery has focused on validated targets

**Figure 1.**
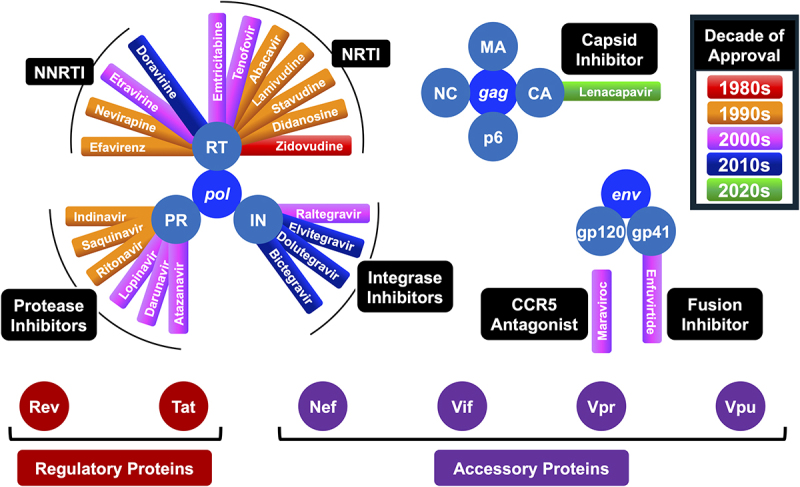
Drug development over time across the HIV proteome.

The discovery of HIV as the etiologic agent of AIDS in the 1980s spurred tremendous research into the development of antivirals, even as HIV spread and progressed into a global pandemic that persists to this day. In the 1990s effective treatment available for people living with HIV and AIDS began to come into focus in the form of antiviral drug combinations, as protease inhibitors began supplementing the drug regimens that targeted reverse transcriptase. As shown in Figure 1, the first decades of HIV drug development targeted the HIV reverse transcriptase and protease as the main focus of drug development in the HIV antiviral space. Over the years, research on these HIV enzymes led to the development of multiple molecules that increased efficacy of the evolving antiviral therapy. This left most of the HIV proteome untouched until introduction of integrase inhibitors became common in antiretroviral therapy, ART [10]. Antivirals that targeted virus fusion and entry are represented by enfuvirtide and the only host-binding antiviral, maraviroc, respectively.

The proteins produced during HIV replication are represented by circles with their corresponding names. Antiviral drugs are noted in color-coded boxes attached to the corresponding target. Each antiviral drug box is filled in with a color corresponding to the decade it was approved. Drug classes of the antiviral drugs are noted in black boxes next to the groups of antivirals.

However, the idea that capsid did not progress from a drug target to an approved antiviral until lenacapavir highlights key points in the overall approach to antiviral drug development with HIV and many other viruses. Reverse transcriptase and protease inhibitors were initially successful targets in HIV antiviral development. These targets benefited from available assays and a clear path to commercialization, which made them attractive to both academia and industry. But that early momentum may have come at the cost of broader exploration of other viral and host targets – not because they lacked potential, but because they did carry more scientific and commercial risk. Success with reverse transcriptase and protease inhibitors may have steered away from the unknown, setting a precedent that limited how antiviral discovery evolved in the decades that followed. Many critical components of the HIV proteome have not had substantial drug development (Figure 1, Regulatory and Accessory proteins) thereby creating a therapeutic landscape where progress has often been incremental rather than transformative.

Additionally, focusing on a few targets leads to a significant concern on the evolution of drug resistance in viruses, where an antiviral could have a decreased lifespan in the history of standard of care [11]. This over-reliance on a limited set of targets has allowed HIV to adapt, rendering some treatments less effective over time. Beyond HIV, circulating Influenza A virus adapted to amantadine and rimantadine, greatly dampening the clinical utility of these antivirals [12]. With only a handful of antivirals in the Influenza virus therapeutic repertoire, there is a need for new targets beyond neuraminidase inhibitors (e.g. oseltamivir, zanamivir) and newer targets like cap-dependent endonuclease inhibitors (e.g. baloxavir marboxil).

Clinically, Lenacapavir has demonstrated strong clinical outcomes in patients with MDR HIV [4,5]. Studies have shown that Lenacapavir can achieve significant viral suppression, even in cases where other drugs have failed. By targeting the HIV capsid – a highly conserved structure – Lenacapavir has also shown promise in reducing the likelihood of resistance development [13].

The continued refinement of drugs targeting these validated viral pathways has played a pivotal role in transforming HIV infection from a fatal disease into a manageable chronic condition. Now, the capsid-targeting mechanism of Lenacapavir offers a new approach. Development of mutations in HIV that blocked Lenacapavir action were often seen with patients that were not taking the drug in accordance or adherence to the therapy plan – this represents a real threat to the longevity of Lenacapavir in the standard of care for HIV-1 infection.

## The case for exploring additional antiviral targets

The HIV genome encodes several proteins that have been heavily researched but that currently have no approved antiviral drug targeting them or their function (See Figure 1). Proteins such as Tat, Rev, Vif, Vpu, and Nef play critical roles in HIV replication and pathogenesis but have had relatively few positive outcomes in drug development. These proteins are involved in processes like transcriptional activation, immune evasion, and the modulation of host cellular pathways. Targeting these underutilized components may well unlock novel strategies for combating HIV and addressing persistent challenges like latency and immune escape. For example, Tat protein stands out as a critical regulator of HIV transcription and latency [14]. Similarly, Nef, a virulence factor protein involved in immune evasion, offers an attractive target for restoring the immune response against HIV by countering its downregulation of host factors such as MHC-I [15].

Targeting viral proteins could also be synergized with targeting many well-studied host proteins that are known to restrict HIV. Proteins like SAMHD1, APOBEC3G, and tetherin are integral to the intrinsic host defenses against HIV [16]. Modulating these factors could provide a dual benefit: enhancing the host antiviral response while reducing development of HIV mutations that could resist these antivirals. This approach represents a paradigm shift in antiviral therapy by harnessing the host’s biology to combat infection. A good example is the relatively low development of resistance against Maraviroc which targets the HIV coreceptor CCR5 [17].

## Challenges and opportunities in antiviral drug development

While the exploration of new targets in HIV therapy holds great promise, it is not without significant challenges. One of the foremost obstacles is the complexity of validating new targets. Unlike well-characterized proteins like reverse transcriptase, newer targets often require extensive research to establish their role in the viral life cycle and their suitability for therapeutic intervention. This process can be time-consuming and resource-intensive, delaying the translation of basic research into clinical applications.

Targeting host factors presents additional difficulties, particularly regarding potential toxicity. Since these proteins are integral to normal cellular functions, their inhibition could lead to unintended side effects. For example, modulating SAMHD1 to enhance its antiviral activity might interfere with its role in nucleotide metabolism, leading to adverse effects, such as inflammatory reactions [18]. Overcoming these toxicity issues requires a delicate balance between drug efficacy and safety. Drug discovery for unproven targets and the translational research required, often face challenges in attracting funding, as pharmaceutical companies and investors prioritize projects or drug candidates with clearer paths to marketability. This creates a cycle where the lack of investment hampers progress, perpetuating medical need and reliance on validated targets.

Despite these hurdles, advances in structural biology and AI-based protein modeling offer opportunities to enhance target identification and rational drug design. Furthermore, embracing interdisciplinary collaboration between virologists, immunologists, and structural biologists has been essential to accelerating innovation. To truly advance the field of HIV therapy and antiviral therapy in general, it is imperative to diversify our approach to drug discovery. Basic research plays a vital role in this drug discovery endeavor by elucidating a deeper understanding of HIV’s interactions with the host cells and the immune system, and the virus’s strategies for persistence providing the foundation for innovative therapies. Moreover, exploring broad-spectrum antiviral strategies that target shared mechanisms across viruses could yield treatments capable of addressing co-infections and emerging viral threats.

While the current complement of HIV antivirals has dramatically improved life expectancy and quality of life for millions, effective treatment and the possibility of curing HIV remains an unmet medical need in our world. Challenges such as global access disparities and the emergence of antiviral resistance remain. Long-acting antiretroviral agents, including lenacapavir, remain limited by issues related to distribution and access. At the same time, the pursuit of a cure through various strategies may ultimately require combination approaches that use novel antiviral or host-directed targets. Drug discovery aimed at underutilized regions of the HIV proteome or host restriction pathways should be viewed as new paths to achieving long-term control, accessibility, and eventual eradication of HIV infection.

In conclusion, Lenacapavir marks a milestone in HIV treatment, demonstrating the power of targeting novel aspects of the HIV replication cycle. Its success highlights the potential of expanding the scope of antiviral drug discovery to other targets in virus lifecycles. Exploring new targets, including underutilized viral proteins and host factors, offers a promising path forward that can unlock potential antiviral therapies to address unmet patient needs, and transform the treatment landscape for HIV and other viral infections.

## Data Availability

Data sharing is not applicable to this article as no new data were created or analyzed in this study. Figure 1 was based on public information and processed in Microsoft PowerPoint. The original file is freely available from the author’s Open Science Framework site (https://doi.org/10.17605/OSF.IO/UHVPM).
